# Thinking without knowing: Psychological and behavioral consequences of unjustified confidence regarding blackjack strategy

**DOI:** 10.3389/fpsyg.2023.1015676

**Published:** 2023-01-26

**Authors:** Eric R. Stone, Andrew M. Parker, Ashley Rittmayer Hanks, Robert C. Swiston

**Affiliations:** ^1^Department of Psychology, Wake Forest University, Winston-Salem, NC, United States; ^2^RAND Corporation, Pittsburgh, PA, United States; ^3^Talogy, Glendale, CA, United States; ^4^Allego, Inc., Waltham, MA, United States

**Keywords:** confidence, risk taking, information search, perceived risk, anxiety

## Abstract

In two studies, we explored potential psychological and behavioral consequences of unjustified confidence, including outcome expectations, anxiety, risk taking, and information search and consideration. Study 1 employed an individual-differences approach to examine how participants’ confidence regarding their knowledge of blackjack strategy, controlling for their actual knowledge, correlated with these hypothesized psychological and behavioral variables. Study 2 manipulated participants’ confidence levels to examine these effects. Across the two studies, greater unjustified confidence led to larger bets (a measure of risk taking) and reduced use of hints designed to improve play (information search and consideration). Unjustified confidence also increased participants’ outcome expectations and lowered anxiety levels. Implications of these findings, such as for educational interventions, are discussed.

## Introduction

“The ultimate contribution of research on confidence for decision-making theory and practice depends on the demonstration of consequences of confidence for decision-relevant behavior” (Sniezek, 1992, p. 144).

This observation, made over 30 years ago, remains relevant today and points to an ongoing challenge for behavioral decision research—to connect our understanding of factors driving decision processes, of which we have abundant evidence, with the psychological and behavioral consequences of those same decision processes, of which we have growing but still sparse evidence (see Weller et al., 2012; Parker et al., 2015). In keeping with the above quotation, our focus here is on confidence, and specifically on the need for both empirical examinations of the various potential consequences of confidence (Alba and Hutchinson, 2000; Parker et al., 2012; Parker and Stone, 2014; Razmdoost et al., 2015) and on the development of a theoretical framework within which to structure these empirical inquiries. Although confidence is a broad concept and can entail many different types of confidence, such as in one’s abilities or performance (e.g., Harvey, 1994; Stone et al., 2011), the focus here is on confidence in one’s knowledge specifically.

Many years of research demonstrate that people’s confidence in their knowledge (sometimes referred to as subjective knowledge) is often not justified by their actual knowledge (Yates, 1990; Alba and Hutchinson, 2000; Moore and Healy, 2008). On tests of knowledge people often think they get more items correct than they actually do (i.e., overconfidence), particularly with tests that are more difficult (e.g., Oskamp, 1965; Fischhoff et al., 1977; Brenner et al., 1996; Klayman et al., 1999).^1^

Indeed, overconfidence is one of the most studied phenomena in the field of judgment and decision making (JDM), and research on overconfidence has been highly influential outside of the JDM field as well (see Moore and Healy, 2008). Given its prevalence, a substantial body of literature has focused on understanding why overconfidence occurs and debiasing techniques to reduce its prevalence. For example, Koriat et al. (1980) showed that one cause of overconfidence is disproportionate attention to reasons why one might be correct vs. why one might be wrong, and showed that an intentional focus on “con” reasons can reduce overconfidence.

Despite the large amount of research demonstrating the prevalence of overconfidence and its causes, until recently there has been relatively little work examining the *consequences* of overconfidence. Instead, judgment and decision-making researchers have typically assumed serious problems can result from confidence, if it is not justified by underlying knowledge. This assumption is understandable, as many actions are based in part on feelings of confidence in knowledge (Griffin and Tversky, 1992; Russo and Schoemaker, 1992). Parker and Stone (2014) referred to these effects as consequences of unjustified confidence, to emphasize (1) that it is feelings of confidence that are driving the effects, and (2) that the effect of confidence is not due to a correlation with knowledge. Typically unjustified confidence effects are examined by computing the partial correlation between confidence and some outcome variable while controlling for actual knowledge (e.g., Jaccard et al., 2005; Parker and Stone, 2014; Bannier and Schwarz, 2018), though sometimes confidence is manipulated (e.g., Hadar et al., 2013; Kuusela et al., 2017), in which case the design of the study provides a test of the causal influence of confidence.

Typically, researchers have assumed that effects of unjustified confidence are negative, limiting the quality of the resulting decisions (e.g., Griffin and Tversky, 1992; Yates et al., 1996; Razmdoost et al., 2015; Moore et al., 2017; Amini et al., 2020; Lindhiem et al., 2020). An alternative argument suggests that confidence (even if unjustified) may have positive effects under certain conditions (see Alba and Hutchinson, 2000; Kahneman, 2011). For example, if confidence serves to increase risk taking, then confidence, even if unjustified, might serve as a corrective factor to combat excessive risk aversion (see Kahneman and Lovallo, 1993). Further, in social situations, the more confidence a person exudes, the more competent he or she may be perceived as being (Fox and Walters, 1986; Cutler et al., 1990; Price and Stone, 2004; Li et al., 2020), potentially increasing persuasiveness. And having confidence in one’s knowledge may lead one to act where action is appropriate, such as undertaking financial planning for retirement (Parker et al., 2012).

Despite this work, however, relatively little research has examined the consequences of confidence, with the vast majority of the work being on predictors of overconfidence rather than on outcomes of overconfidence. Further, most of the work that has been done has explored effects in isolation, such as on anxiety or information search, which makes it difficult to determine if any apparently contradictory effects (such as confidence is helpful in reducing anxiety but detrimental in decreasing information search) are due just to different domains being investigated. Thus, there is a need for systematic investigations that create a more complete picture of the potential impacts of confidence. Here we look at four such consequences simultaneously, which allows us to examine (1) the effects on each consequence with the same experimental materials, and (2) the relationships among the consequences.

Another potential concern in this line of research is that if confidence does reflect underlying knowledge, effects of the two may be confounded. Parker and Stone (2014) examined two methodological approaches for determining the relationship between confidence, and its correspondence or lack of correspondence with knowledge, with variables hypothesized to be influenced by confidence. For most situations, they suggested using an “unjustified confidence” approach, where the consequence is predicted by confidence controlling for knowledge. This paper builds off the insights from that paper and other work examining consequences of confidence (e.g., Hadar et al., 2013; Razmdoost et al., 2015). Finally, there is a need to incorporate stronger methods for establishing the causal influence of confidence. We do this in Study 2, described below, using an experimental manipulation of confidence.

In the sections that follow, we review existing evidence on the consequences of confidence, and specifically unjustified confidence, leading to specific hypotheses. We then proceed to test these hypotheses in two studies. We close with implications of these results, both practically and for a larger conceptual model, as well as provide a roadmap for future research mapping out diverse consequences of confidence.

## Unjustified confidence as a predictor

For the current study, we focus on two psychological consequences, outcome expectations and state anxiety, and two behavioral consequences, risk taking and information search and consideration.

### Psychological consequences

#### Outcome expectations

Following from Social Learning Theory (e.g., Bandura, 1977), we define outcome expectations as the assessment of expected gains and losses associated with a behavior or undertaking. To our knowledge, most of the work that has examined the effect of confidence on outcome expectations has focused on losses, i.e., perceived risk. When one can use one’s knowledge to decrease risk, there should generally be a negative relationship between confidence in one’s knowledge and perceived risk. For example, controlling for objective knowledge and other variables, Sharifpour et al. (2014) found that greater subjective knowledge decreased the perceived risks involved in tourism. Similarly, Klerck and Sweeney (2007) found that, for objectively knowledgeable consumers, subjective knowledge decreased the perceived physical risk associated with genetically modified food.

However, Klerck and Sweeney (2007) also found that the negative relationship between subjective knowledge and perceived risk only held when objective knowledge was high; when objective knowledge was low, increased subjective knowledge was associated with *increased* risk perceptions. The authors suggested this interaction occurred because the determinants of subjective knowledge (e.g., media reports) were generally negative, so that when (low objective knowledge) participants were relying primarily on these negative sources of information, their risk perceptions increased. This interpretation is consistent with a more recent study on waterpipe tobacco use by Lipkus et al. (2014), who found that greater perceived knowledge was associated with a greater perceived risk of harm if one continued to smoke. Presumably, this relationship occurred because the sources of information that produced high subjective knowledge were negative about waterpipe use, and there was no obvious way to use one’s knowledge to develop more positive outcome expectations (see also Melki et al., 2022). If instead one’s perceived knowledge can be used to produce positive outcomes, then greater confidence in one’s knowledge should lead to better outcome expectations, as in Sharifpour et al.’s (2014) study. We focus on this type of situation in the current work.

#### State anxiety

Many researchers, across a wide range of subject areas and operationalizations of confidence, have posited that feelings of confidence reduce the experience of anxiety. For example, a key component of Harter’s (1981) developmental model of mastery motivation is that perceived competence reduces anxiety levels. In keeping with this idea, sports psychologists have found a negative relationship between confidence in one’s ability and experienced anxiety while performing (e.g., Voight et al., 2000; Kjørmo and Halvari, 2002), high-confidence social workers and students reported feeling lower levels of on-task anxiety than did their low-confidence counterparts (Bogo et al., 2017), perceived knowledge of computers was strongly and negatively related to anxiety regarding computer use (Anderson, 1996), confidence in one’s coping ability predicted decreased levels of later state anxiety above and beyond trait anxiety (Zalta and Chambless, 2012), individuals’ confidence levels regarding tests performance is negatively related to their experienced anxiety when taking tests (Stankov et al., 2012), and specific self-confidence in consumer decision making (i.e., confidence regarding the product under consideration) negatively influenced state anxiety regarding decision making (Keng and Liao, 2013). Thus, there is substantial evidence for a link between confidence and anxiety in a wide variety of situations (though see Jiang and Kleitman, 2015).

Nonetheless, there are at least two reasons for caution in concluding that unjustified confidence reduces state anxiety. Although there are some exceptions (e.g., Davey et al., 1996), most of the work investigating confidence and anxiety has been correlational. Thus, the direction of the relationship is unclear, and indeed there is some research suggesting that anxiety produces low confidence, rather than the reverse (George, 1994; Gino et al., 2012). Furthermore, virtually none of the previous work has manipulated confidence or controlled for objective knowledge or ability; thus, it is unclear if confidence is producing changes in anxiety or if actual knowledge or ability is producing these changes. One exception is a study by Davey et al. (1996), who manipulated confidence *via* false feedback on a previous problem-solving task. Participants in the decreased confidence condition exhibited greater anxiety and catastrophic worrying in comparison to participants in the increased confidence condition.

### Behavioral consequences

#### Risk taking

Risk taking involves an action undertaken to receive some desired outcome at the risk of some less-desired outcome. Many researchers (e.g., Dunning et al., 1990) have argued that high confidence in one’s assessments should make it more likely that one will take risks and less likely that one will undertake insurance measures to minimize them. The basic idea is that confident decision makers believe they can use their knowledge to achieve favorable results from their risk taking (March and Shapira, 1987). This association may result from people being more willing to take risks in areas in which they know more (Heath and Tversky, 1991; Hadar et al., 2013) and being more apt to avoid risks where their perception of risk is high (Lopez-Quintero and Neumark, 2010). Further, heightened anxiety leads people to selectively attend to negative outcomes (Mathews and MacLeod, 1994) and thus to more risk-averse decisions (Raghunathan and Pham, 1999; Maner et al., 2007; Broman-Fulks et al., 2014). Regardless of the precise mechanism, there is good reason to believe that increasing confidence will lead to increased risk taking.

Indeed, a substantial body of research supports this prediction. Much of this work has been conducted in the financial domain, where a growing body of research shows that increased subjective knowledge increases willingness to take investment risks (Hadar et al., 2013; Allgood and Walstad, 2016; Larson et al., 2016). Work outside the financial domain has operationalized confidence in a number of different ways, making the work harder to summarize (see Gloppen et al., 2010). Nonetheless, the existing evidence suggests that the confidence—risk taking relationship holds more generally (e.g., Shou and Olney, 2021). In the health domain, Parker and Stone (2014) showed that greater unjustified confidence among adolescents is associated with marijuana use. Similarly, Jaccard et al. (2005) found that adolescent perceived knowledge about birth control, controlling for actual knowledge, was positively associated with becoming pregnant (a potential consequence of greater risk taking). Still, there is some evidence that this positive relationship may not hold in all situations (e.g., Browne, 1989; Melki et al., 2022), and more work outside the financial domain would help test the robustness of this relationship.

#### Information search and consideration

Lack of information search and consideration is frequently theorized as one of the effects of high confidence (e.g., Dunning et al., 1990; Alba and Hutchinson, 2000; Renner and Renner, 2001; Kahlor, 2010; Hadar and Sood, 2014; Razmdoost et al., 2015; Chin and Williams, 2020), presumably due to a perceived lack of need. For example, Kahlor’s (2010) Planned Risk Information Seeking Model (PRISM) states that a primary driver of information seeking is perceived knowledge insufficiency (see also Hovick et al., 2014). From this perspective, individuals with high confidence in their knowledge should conduct less thorough information searches and also consider available information to a lesser extent than less confident individuals. In effect, if one is confident about his or her knowledge, what more does he or she need to know? In contrast, if one is not confident, then seeking out and using external sources of information feels more necessary, in part to reduce perceived risk (Klerck and Sweeney, 2007; Huurne and Gutteling, 2008; Zhao and Cai, 2009; Gutteling and de Vries, 2017) and feelings of anxiety (Raghunathan and Pham, 1999; Zhao and Cai, 2009; Lee and Hawkins, 2016).

Empirical support for the confidence—information search link comes from a variety of research areas. For example, Chin and Williams (2020) found that consumers with greater subjective knowledge were less likely to use a home-buying and mortgage education website, and, when they do, use it for less time. Radecki and Jaccard (1995) found that higher perceived knowledge was related to acquiring less information about nutrition and birth control through an Informational Display Board. Kuusela et al. (2017) found that subjective knowledge was negatively related to amount of effort exerted, as operationalized by engaging in information processing operations when selecting a residential insurance contract. Kim et al. (2010) showed that overconfident students worked on fewer practice problems than did better calibrated students. Confidence in one’s knowledge reduces one’s willingness to take advice as well, whether the advice was from another participant in the study (Gino et al., 2012), from dealer opinions in purchase decisions (Brucks, 1985), or from helpful actuarial judgment aids (Sieck and Arkes, 2005; though see Bishop and Barber, 2012).

Note that there is other work suggesting a positive relationship between confidence and information search and consideration (for discussions, see Jiang and Rosenbloom, 2013; Kuusela et al., 2017; Utkarsh et al., 2019). This finding appears to hold when other types of confidence are being considered, such as confidence in one’s ability to use information sources well or not to be manipulated by misleading sources (Loibl et al., 2009; see also Kahlor, 2010). Thus, when other types of confidence are considered, confidence may be positively related to using external sources of information (Locander and Hermann, 1979; Jiang and Rosenbloom, 2013; Allgood and Walstad, 2016; Utkarsh et al., 2019). For the purpose of the current paper, we are restricting our investigation to confidence in one’s factual knowledge specifically, so expect a negative relationship between confidence and information search and consideration to occur.

## The present research

The aim of the current research is to investigate the impact of unjustified confidence on the above psychological and behavioral consequences in one study. So doing allows us to examine the effects on each consequence within the same context as well as to investigate the interrelationships among the consequences. Blackjack provides a useful domain for studying these questions for a variety of reasons. It is sufficiently simple that the game can be played in a laboratory, with tight control while maintaining the key elements of the real-world game. Blackjack also provides a context in which each of these variables are easily measured. For example, in blackjack the amount of money bet is a relatively clear and unambiguous indicator of risk taking, whereas in other domains (e.g., stock market investing) there is more disagreement regarding what risk entails (see Bodie et al., 2002). Note that the focus of our work is not on understanding blackjack play and betting *per se*; there already exist many excellent accounts of blackjack behavior, e.g., Wagenaar’s (1988) detailed discussion of cognitive illusions influencing blackjack gambling. Instead, the aim of our work is to understand the effects of confidence specifically, and blackjack is being used as the context to investigate this issue.

Based on our literature review we have developed the following four hypotheses, which are tested correlationally in Study 1 and causally in Study 2:

*H1*: **Higher levels of unjustified confidence will increase positive outcome expectations**. Because participants can use their knowledge (at least in theory) to improve their play, thinking that one knows more should produce a stronger belief that the game will go well.

*H2*: **Higher levels of unjustified confidence will decrease state anxiety**. Similar to other domains studied, being confident in one’s blackjack knowledge should reduce feelings of anxiety while playing blackjack.

*H3*: **Higher levels of unjustified confidence will increase risk taking**. Because participants can use their knowledge to improve their play, thinking that one knows more should lead one to be more risk taking while playing.

*H4*: **Higher levels of unjustified confidence will decrease information search and consideration**. Participants who believe they are knowledgeable will see less need for additional information to help them play, so will be less likely to seek out or use that information.

We investigate these hypotheses in two studies. Study 1 takes a correlational approach focused on individual differences, whereas Study 2 takes an experimental approach to assess causality.

## Study 1

### Method

#### Study overview

The study consisted of two sessions. In Session 1, participants completed a questionnaire designed to assess their knowledge of casino blackjack rules, as well as a measure designed to assess their knowledge of and confidence regarding blackjack strategy. Only those participants who demonstrated a working knowledge of casino blackjack rules were invited back for the second session. In Session 2 we simulated a casino blackjack game, where participants played as if they were in an actual casino. Our primary interest was in the relationship between confidence, controlling for knowledge (both assessed in Session 1), with measures of outcome expectation, anxiety, risk-taking behavior, and information search and consideration (all assessed in Session 2).^2^ The full questionnaire measures used in both studies are provided in Supplementary material (pp. 5–27).

#### Participants

One hundred and forty-nine introductory psychology students participated in Session 1 as a means of fulfilling a course requirement. Students were instructed to sign-up for the study only if they were familiar with casino blackjack and were provided a test of casino blackjack rules during Session 1. Participants who answered 60% correct or greater were eligible to participate in Session 2. One participant was eliminated due to excessive non-response on a different questionnaire taken in Session 1. All subsequent analyses are based on the remaining 118 (72 male, 46 female) participants (79% of initial sample).

#### Test of casino blackjack rules

Ten items assessed knowledge of the basic rules of casino blackjack. Sample items include “How many points is a face card (King, Queen, or Jack) worth?” and “What does it mean for a player to *split* his or her hand?” Respondents who answered six or more questions correctly were asked to participate in Session 2.

#### Knowledge-confidence assessment

This questionnaire measured participants’ knowledge and associated confidence levels regarding optimal blackjack strategy. The Knowledge-confidence assessment (KCA) was comprised of 40 items covering four areas of blackjack play: when to hit versus stand, split, double down, and buy insurance. All items presented the player’s hand and the dealer’s upcard and asked participants to determine the correct play “to maximize your earnings.” More specifically, for each blackjack scenario, participants first judged which play (e.g., hit or stand) was correct (a deterministic judgment) and then, second, judged how confident they were that their answer was the correct choice (a likelihood judgment). A sample item was:

**Table:** 

1	**You**		10, 7	**Dealer**	10	
	To maximize your earnings, should you?: Hit or Stand.					
	50%	60%	70%	80%	90%	100%
just guessing				absolutely sure		

The actual items were chosen *via* a pseudo-random procedure, where we first eliminated all situations deemed “obvious” (e.g., whether you should hit or stand if your point total was 20). Next, we constrained the remaining possible hands to cover as broad a range of possible items as possible (e.g., we asked about whether one should split aces once but only once), randomly selecting from the remaining possibilities. We scored correctness according to whether participants followed “basic strategy” (Shackleford, n.d.; see also Thorp, 1966) and then computed participants’ average confidence and percentage correct.

#### Psychological and behavioral consequences

##### Outcome expectations

We asked participants (1) “What is the likelihood that you will win more than the average person during today’s blackjack game?” (0%–100%) and (2) “What is the likelihood that you will win more money than you lose during today’s blackjack game?” (0%–100%). The two items were highly correlated (*r* = 0.73), so we averaged the two to form our overall measure.

##### Anxiety

The State–Trait Anxiety Inventory (STAI; Spielberger et al., 1970) is a widely used and reliable (Barnes et al., 2002) measure of anxiety. The participant’s state anxiety score was the mean response across the 20 questions that comprise the STAI (e.g., “I was tense”). We modified this scale to ask about feelings during the blackjack game; specifically, participants rated each of the items according to “how you felt while playing blackjack, that is, during today’s experiment” on the response scale: (1) almost never, (2) sometimes, (3) often, (4) almost always. Cronbach’s alpha for our study was 0.93, demonstrating high reliability for our modified version of the STAI.

##### Risk taking

As described below, respondents were allowed to place hypothetical bets between $10 and $100 for each of 20 hands of blackjack (they were required to bet at least $10 on each round). Risk-taking behavior was defined as the participant’s average bet across these 20 hands.

##### Information search and consideration

We gave participants the opportunity to view “hints for blackjack play.” These hints were based on basic blackjack strategy and were designed to improve participants’ play. Sample hints included, “When *you* have 12–16, stand when *dealer* has a 6 or lower showing” and “Always split A’s and 8’s.”

At the conclusion of 20 hands of blackjack, participants were asked nine questions designed to measure the extent to which they used the hints and would want additional information if playing casino blackjack in the future. Sample items are “How often did you look at the blackjack suggestions?” and “Would you want to consult a book or person for additional suggestions on strategy?” The nine items were first reverse scored, as necessary, so that higher scores indicated greater degrees of information search and consideration, and then z-standardized due to the use of multiple response scales. One of the potential items on the questionnaire produced an item-total correlation of −0.12 in the direction opposite our expectation and was eliminated^3^; the mean of the remaining eight standardized responses comprised our measure of information search and consideration (Cronbach’s α = 0.85).

#### Procedure

##### Session 1

Upon arriving at the session, participants first completed the test of casino blackjack rules. Although participants needed to do reasonably well on this test to be eligible to take part in the next session, we provided a brief (4 min) overview of casino blackjack rules and play to ensure that eligible participants knew all the rules. Following the overview, participants completed the KCA, and eligible participants were encouraged to come back to play blackjack in Session 2.

##### Session 2

Session 2 was conducted with groups of four (or fewer) participants and took approximately 1 h to complete. Upon arriving at this session, participants first completed a questionnaire to assess their outcome expectations regarding the upcoming blackjack play. Next, the researcher explained that participants would play 20 hands of casino blackjack and briefly reviewed the rules that applied. The casino blackjack game was set up to mimic an actual casino experience as closely as possible. A dealer sat at a table facing the players and dealt standard casino cards from a card shoe, as in an actual casino game. All participants were given $800 worth of chips and instructed that they must bet between $10 and $100 on every hand. To encourage participants to play realistically, as if playing with their own money, they were informed that one participant (selected at random) would receive 10% of his or her final amount in cash. Additionally, participants were provided with a sheet of paper that contained the “hints for blackjack play.” This sheet was placed on their desk so that they could access it whenever desired. Participants were told that following the hints would improve blackjack play in most situations, but they were allowed to play in whatever manner they desired.

Because performance in blackjack is highly dependent on many random factors, we were concerned that these random factors would have an undue influence on our dependent measures. For example, a participant who played against a dealer who had bad hands would win more frequently, likely leading them to bet more, feel less of a need to use the hints, etc. Thus, we included a number of control procedures with the aim of equating these random factors to the extent possible. Dealers’ hands were randomly constructed and ordered prior to the study, and that set of hands was used for every participant. These cards were dealt from a shoe that was designated for the dealer. Thus, all participants across all sessions played against the same dealers’ hands. Participants’ cards were dealt from a separate shoe, which contained the rest of the cards in a random ordering that varied from participant to participant (controlling the player cards dealt was not possible, due to choices players made during the game). This procedure was explained to participants and it was stressed that the dealers’ cards were not “stacked” for or against the players. Furthermore, the players were assured that their cards were dealt at random, as they were. To promote independent observations, participants were seated at side-by-side desks (i.e., in a row) separated by partitions. Therefore they could not see each other (or each other’s cards or bets), yet all could see the dealer’s cards. After 20 hands, participants completed a final questionnaire including the modified version of the STAI, the questions designed to measure participants’ information search and consideration, as well as additional items included for exploratory purposes.

#### Analytic approach

The primary goal of our analyses was to test whether confidence, when not justified by actual knowledge, would correlate with the consequence variables as indicated in Hypotheses 1–4. As discussed by Parker and Stone (2014), when one’s primary interest lies in the effect of unjustified *confidence*, as in the present research, the preferred analytic technique is the semi-partial correlation between confidence and the outcome measure controlling for percent correct (referred to as unjustified confidence). Thus, we report that analysis throughout. Although we have directional hypotheses for the relationships between unjustified confidence and the four psychological and behavioral variables, we conducted two-sided tests in recognition of the conflicting results in the literature regarding some of the outcome variables. We also examine the correlations among the psychological and behavioral variables in a more exploratory fashion.

### Results

On average, participants gave confidence judgments of 80.1% (sd = 7.37) on the KCA, but were only correct 62.0% (sd = 9.50) of the time. Thus, participants were overconfident, on average, by 18.1% (sd = 10.10). Indeed, 114 out of the 118 participants exhibited some degree of overconfidence, and 94 were overconfident by at least 10%. In addition, and as is often the case (Alba and Hutchinson, 2000), confidence and knowledge were positively correlated but only modestly so, r = 0.30, *p* < 0.001.

There were strong relationships between unjustified confidence and the psychological variables, such that those with greater unjustified confidence had greater outcome expectations for winning at blackjack (semi-partial r = 0.30, *p* < 0.001) and reported lower anxiety (sr = −0.35, *p* < 0.001). The relationships between unjustified confidence and the behavioral measures were in the expected direction but somewhat weaker, such that those with greater unjustified confidence demonstrated less information search and consideration (sr = −0.23, *p* = 0.02) and more risk taking (sr = 0.13, *p* = 0.17), though the latter relationship did not reach significance.

There were substantial relationships among several of the psychological and behavioral variables (Table 1). In particular, participants with greater outcome expectations bet more than did participants with less positive expectations, *r* = 0.39, *p* < 0.001; and more anxious participants bet less than did less anxious participants, *r* = −0.25, *p* = 0.007. There was also a large positive correlation between anxiety level and information search and consideration, *r* = 0.31, *p* = 0.001. The associations among the other variables were not significant.

**Table 1:** Correlations among the psychological and behavioral consequences in Study 1.

	Anx.	Avg. bet	ISC
Outcome expectations	−0.12	0.39^***^	−0.13
Anxiety	−	−0.25^**^	0.31^***^
Average bet		−	−0.12
Information search and consideration			−

^**^p < 0.01, ^***^p < 0.001.

### Discussion

The results of Study 1 mostly supported our hypotheses. In particular, unjustified confidence was related to higher outcome expectations (H1), reduced anxiety (H2), and less information search and consideration (H4). Unjustified confidence was also associated with greater risk taking (H3), but not significantly so. We also saw significant correlations among the various consequences, and in particular between the psychological variables with the behavioral ones. These results, however, are purely correlational, so do not provide strong causal evidence. In addition, our behavioral measure of information search and consideration was a self-report measure of behavior, rather than a measure of behavior itself. In Study 2, we manipulated confidence in order to provide stronger causal evidence of the effects of confidence and modified some of our measures to more fully capture the constructs under investigation.

## Study 2

Our primary goal of Study 2 was to manipulate the level of unjustified confidence and examine the effects of this manipulation on the components of our model. Overconfidence can be difficult to influence (e.g., Sieck and Arkes, 2005), so to provide the best chance of manipulating confidence (and hence overconfidence), we included two treatments. The first treatment was designed to reduce confidence without influencing knowledge, and the second was designed to raise confidence without influencing knowledge. The aim of this work was not to examine the impact of each of these particular treatments, but to produce a situation where participants had higher confidence in one condition and lower confidence in another condition. Using these two treatments (rather than only using one designed to raise or lower confidence) should provide a more powerful manipulation than only including one treatment.

In addition, rather than having participants play in a simulated casino with a fixed shoe, we constructed a blackjack computer program. This procedure facilitated individual testing and the collection of more data per participant, which allowed all portions of the game to be randomized rather than having the dealer’s hands set in advance. The greater number of hands also provided a more reliable measure of risk taking, which had produced the weakest results in our first study. Finally, the use of the computer program allowed for more objective measurement of how frequently (and how long) participants accessed the hints for blackjack play, which were implemented through a separate, pop-up computer screen.

### Method

#### Participants

One hundred and fifteen introductory psychology students participated as one means of partially fulfilling a course requirement. All participants needed to pass the test of casino blackjack rules, which was completed during a mass testing session.

#### Measures

##### Test of casino blackjack rules

This 10-item measure was a revised version of the test used in Study 1, replacing one non-discriminant item. Because the test was now more difficult, we only required that participants answer 50% of the questions correctly to be eligible to participate.

##### Knowledge-confidence assessment

This study used the KCA questionnaire that was used in Study 1, as well as a second version designed to check the effectiveness of the confidence manipulations.^4^ Order of the two versions of the KCA was counterbalanced within each session, so that an approximately equal number of the participants in each session got each version of the assessment first.

##### Outcome expectations

This measure was modified from Study 1 to assess the construct more thoroughly. In particular, rather than asking just about likelihood of winning, this questionnaire asked questions regarding both likelihood of winning and likelihood of losing on a 0 to 100% scale, as well as how one would feel if one won or lost on a 1–7 scale (i.e., both likelihood and utility). For participants whose self-reported probability of winning was not equal to one minus the self-reported probability of losing (maximum discrepancy of 0.3), we proportionately recoded their probability judgments so that they were forced to sum to 100%. We then calculated gain expectation as the product of likelihood and utility of winning, and loss expectation as the product of likelihood and (dis)utility of losing. Our overall measure of outcome expectations was computed by subtracting loss expectation from gain expectation.

##### Anxiety

The modified version of the STAI used in Study 1 was also used in this experiment to measure participants’ anxiety levels during blackjack play. However, to better reflect the amount of anxiety they experienced during the game, we modified the response scale to: (1) not at all, (2) somewhat, (3) moderately so, or (4) very much so.

##### Risk taking

As in Study 1, the measure of risk-taking behavior was the participant’s average bet across the total number of rounds of blackjack played.

##### Information search and consideration

The behavioral measure of information search and consideration was the frequency and duration of participant’s consultation of the “hints for blackjack play.” These hints were identical to those used in Study 1, except they were provided through a computer pop-up screen rather than being given on a sheet of paper. Because both the number of times and the number of seconds viewed were positively skewed, the natural log of each (plus one, to account for the many zero responses) was computed, and the two scores were standardized and averaged to comprise the behavioral information search and consideration score.

We also included a self-report measure, similar to that from Study 1. However, due to the different method by which the hints were displayed, the self-report questionnaire was modified slightly, becoming a seven-item questionnaire. We eliminated the same item as in Study 1, as it again had a negative item-total correlation. Our final self-report measure included six items; Cronbach’s alpha was 0.87. The behavioral and self-report measures were highly correlated (*r* = 0.76), so we averaged the two measures to produce our overall measure of information search and consideration.

#### Casino blackjack game

The C++ computer program created for this experiment is a standard one-player blackjack computer game, based on the rules of blackjack as played in most casinos. All players started with $5,000. For each round, the player was first asked to place a bet between $1 and $100. Then, participants made decisions in whatever manner they desired. The “blackjack hints” were available for the player to view at any time during play. Players played for the maximum amount of time possible given the time constraints of the experiment (up to 30 min). Prior to actual play, players played five practice rounds, which were not recorded.

#### Experimental manipulation

The experiment was conducted with groups of six (or fewer) participants and took approximately 2 h to complete. Each group was assigned to one of two experimental conditions, whereby approximately half of the participants (*n* = 59) received the manipulation designed to decrease confidence (the *lower-confidence* group) and the other half (*n* = 56) received the manipulation designed to increase confidence (the *higher-confidence* group). We varied the experimental condition between sessions rather than within a session due to the logistical difficulty of conducting both manipulations within the same session. Particular care was taken to standardize the instructions across sessions.

##### Lower confidence

This manipulation involved providing participants with personalized feedback regarding their calibration performance on the initial knowledge-confidence assessment. Specifically, each individual’s responses on the KCA were recorded and his or her personalized calibration graph was constructed from the responses. A calibration graph shows the percentage of correct choices as a function of each specific confidence judgment category (see Yates, 1990). For example, if a given respondent was correct 85% of the time that he or she used the 100% confidence judgment category, this fact would be displayed pictorially on the calibration graph.^5^ The experimenter conducted a brief (approximately 1 min) one-on-one feedback session with each participant. This session included an explanation of the personalized calibration graph and suggestions for improvement, with particular emphasis placed on use of the extreme (0% and 100%) judgment categories (see the Supplementary material, p. 3, for more detail).

In previous studies (e.g., Lichtenstein and Fischhoff, 1980; Stone and Opel, 2000; Sieck and Arkes, 2005), similar manipulations have decreased overconfidence by decreasing confidence—that is, the calibration feedback leads to lower (more realistic) confidence judgments. Note that although we used this manipulation to decrease confidence, the actual purpose of the calibration feedback is to improve calibration. Thus, participants were given suggestions for calibration improvement, which typically (but not always) included the suggestion to make lower confidence judgments (because 51 of the 59 participants in this condition were overconfident). Further, because the judgment categories reflective of certainty or near-certainty tend to have the poorest calibration (Fischhoff et al., 1977), particular emphasis was placed on avoiding the use of these judgment categories, when appropriate.

At the conclusion of the one-on-one feedback session, the experimenter asked each participant to restate the calibration advice given to him or her. After all participants in the group had received the one-on-one feedback, the experimenter gave each participant his or her calibration graph to review for approximately 2 min, after which it was returned to the experimenter.

##### Higher confidence

This manipulation involved providing participants with blackjack content information that appeared to be helpful, but actually was quite difficult to use to improve casino blackjack play. The information was presented as a graph of “The odds of possible outcomes, depending on the player’s hand.” In particular, for each hand count (e.g., 11), the graph provided the chances of busting, having a hand between 17 and 21, and having a hand less than 17 if the participant drew an additional card. Knowing these odds should make participants feel more knowledgeable about blackjack, but should not actually improve their play because it was not clear how to use this knowledge. Thus, much in the same way that participants in Oskamp’s (1965) study felt more confident as a result of the information presented to them, leading to increased overconfidence, we hypothesized that our participants would become more overconfident (see also Stone and Opel, 2000).

The experimenter conducted a brief (approximately 1 min) one-on-one session with each participant, which included an explanation of the outcome odds graph and encouragement that this information should be helpful when making judgments regarding blackjack play strategy. After the graph was explained, each participant was given the opportunity to ask questions and received a copy of the blackjack information graph to review for approximately 2 min, after which it was returned to the experimenter. (See the Supplementary material, pp. 3–4, for more detail.)

#### Procedure

At the beginning of each experimental session, the experimenter first gave a short lecture (approximately 4 min) on casino blackjack rules, like that given in Study 1. Following this lecture, the experimenter distributed the first KCA. To encourage participants to attend closely to the task, they were told that the person who performed best would be rewarded with 20 dollars. “Best performance” was defined as a combination of the highest percent correct and best calibration. Because appropriate confidence judgments (i.e., well-calibrated judgments) were encouraged, the experimenter gave a brief lecture on the concept of calibration as well.

After completing the KCA, participants completed a filler task and an additional questionnaire used for exploratory purposes. While participants completed those measures, the KCA responses of participants in the lower confidence condition were scored by the experimenter and a research assistant, and the calibration graphs were constructed for use during the feedback session in the lower confidence condition.

Then, depending on condition, the group of participants received the relevant confidence manipulation. As motivation to attend to the information, participants were told that that they would be filling out a second KCA and that the participant with the best performance would also be rewarded with 20 dollars (we, in fact, rewarded the best performance in each condition).

After completing the second KCA, participants completed the outcome expectations measure. Then, the experimenter gave instructions regarding the blackjack computer program and blackjack play. Participants were encouraged to play blackjack realistically. As incentive to play realistically, participants were informed that three of them (selected at random) would be rewarded with a small fraction of his or her final amount in cash. The grand prize was 2% of the final amount; if a participant ended the game with $5,000, for example, his or her grand prize would have been $100.

Unforeseen to us, the manipulation designed to decrease participants’ confidence took slightly longer on average than did the manipulation designed to increase participants’ confidence, and substantially longer in a couple of sessions (e.g., computer problems creating the calibration graphs). Because of time constraints regarding how long the participants were available, lower-confidence participants (*M* = 110.8) thus, on average, played fewer rounds of blackjack than did higher-confidence participants (*M* = 141.5), *t*(112) = 3.96, *p* < 0.001. Consequently, lower-confidence participants had fewer opportunities to place bets, to consult the blackjack hints, etc.

To address this problem, two steps were taken. First, participants who played fewer than 60 rounds of blackjack, which was one-and-a-half standard deviations below the mean number of rounds played, were excluded from all analyses (*n* = 7, all from the lower-confidence condition). Excluding these participants ensured that the remaining results were based on a reasonably large amount of data (i.e., 60-plus rounds, at least three times the number as in Study 1) and were thus fairly reliable. For the remaining participants, we analyzed only the first 60 rounds of data on the relevant blackjack measures. This approach reduced the power of our study, by decreasing the reliability of our behavioral measures (because there were fewer opportunities to bet and use the hints), but assures that there is an equal amount of data per participant, and thus per condition.

Finally, after playing casino blackjack on the computer, participants filled out a set of additional measures that included the measures of anxiety and information search and consideration.

#### Analytic approach

We first tested the effect of the confidence manipulations on confidence. Because our aim of this manipulation check was to see if there was a differential change from pre-manipulation confidence to post-manipulation confidence depending on condition, we used a repeated-measures ANOVA to examine this question. We ran an analogous ANOVA on knowledge to ensure that knowledge was not differentially influenced.

Our main hypothesis tests were conducted as independent-samples *t*-tests on each of the consequence variables. As our hypotheses are directional and the results from the first study were all in the direction of our hypotheses, these tests are one-sided. As in Study 1, we also examined the correlations among the psychological and behavioral variables, in a more exploratory fashion, with two-sided tests. Finally, to provide a better overall sense of the relationships among the consequence variables across the two studies, we used the meta-analytic procedure recommended by Goh et al. (2016) for combining results of studies within a manuscript to provide overall estimates of the relationships.

### Results

#### The knowledge-confidence assessment

In keeping with the results of Study 1, we observed a substantial amount of overconfidence. On average, participants gave confidence judgments of 78.3% on the initial KCA, but were only correct 62.8% of the time, thereby demonstrating a mean overconfidence of 15.5%. In fact, only 10 of the 115 participants were underconfident. Additionally, the correlation between average confidence and percent correct was even more modest in this study, *r* = 0.13.

As a manipulation check, we conducted a repeated-measures ANOVA on participants’ mean confidence judgments (across the 20 KCA items), with between-subjects factors of manipulation condition and KCA version order and the within-subjects factor of round (first or second time taking the KCA). A highly significant two-way condition by round interaction showed that our manipulation was successful, as the change in mean confidence was different for the two manipulation conditions, *F*(1, 111) = 67.06, *p* < 0.001. Follow-up simple-effect tests showed that the lower-confidence manipulation did significantly decrease confidence from round 1 (*M_1_* = 0.78) to round 2 (*M_2_* = 0.72), *t*(111) = 10.37, *p* < 0.001, one-tailed. Similarly, the higher-confidence manipulation increased confidence from round 1 (*M_1_* = 0.78) to round 2 (*M_2_* = 0.79), although this increase did not reach significance, *t*(111) = 1.32, *p* = 0.09, one-tailed. An analogous repeated-measures ANOVA on percentage of items answered correctly found there were no significant effects of condition on percentage correct (all *p*’s > 0.05), suggesting we succeeded in our goal of manipulating confidence without affecting the percentage of items answered correctly.

#### Effects of manipulated confidence

##### Outcome expectations

Participants in both conditions assessed expected gains to be greater than expected losses, as indicated by a positive mean value of outcome expectations. Nonetheless, as predicted (H1), the higher-confidence participants had a more positive outcome expectation (*M* = 1.69) than did lower-confidence participants (*M* = 0.81), *t*(105) = 3.55, *p* < 0.001.

##### Anxiety

As predicted (H2), higher-confidence participants were less anxious during blackjack play (*M* = 1.89) than were lower-confidence participants (*M* = 2.06), *t*(101) = 1.81, *p* = 0.04, one-tailed.

##### Risk taking

As predicted (H3), participants bet more on average in the higher-confidence condition (*M* = $67.02) than in the lower-confidence condition (*M* = $57.21), *t*(105) = 1.79, *p* = 0.04, one-tailed.

##### Information search and consideration

As predicted (H4), participants’ information search and consideration was greater in the lower-confidence condition than in the higher-confidence condition, *t* (92) = 1.86, *p* = 0.03, one-tailed. The effect was equally strong on the behavioral and self-report measures (one-tailed *p*’s = 0.06 and 0.04, respectively).

#### Relationships among the consequences

The correlations among the consequences were generally in the same direction as in Study 1 (Table 2), but were smaller and less significant (all *p*’s > 0.05). To provide a better overall estimate of the strength of the relationships across the two studies, we used the meta-analytic procedure recommended by Goh et al. (2016) for combining results of studies within a manuscript (see the Appendix for details). Combined across the two studies, participants with higher outcome expectations bet more than did participants with less positive expectations, *r* = 0.28, *z* = 4. 10, *p* < 0.001, and exhibited a trend to consider less information, *r* = −0.11, *z* = −1.58, *p* = 0.11. More anxious participants bet less than did less anxious participants, *r* = −0.19, *z* = −2.69, *p* = 0.007, and considered more information, *r* = 0.23, *z* = 3.21, *p* = 0.001. There was also a marginally significant negative relationship between outcome expectations and anxiety, *r* = −0.13, *z* = −1.89, *p* = 0.06. Average bet and information search and consideration were unrelated, *r* = −0.04, *z* = −0.50, *p* = 0.62.

**Table 2:** Correlations among the psychological and behavioral consequences in Study 2.

	Anx.	Avg. bet	ISC
Outcome expectations	−0.13	0.15	−0.09
Anxiety	−	−0.11	0.12
Average bet		−	0.06
Information search and consideration			−

### Discussion

The results of this study provide causal support for all four of our hypotheses, in that our manipulation of confidence influenced both of the psychological variables, outcome expectations and anxiety, as well as the behavioral variables of risk taking and information search and consideration. Thus, by changing one’s confidence, his or her thoughts and actions will be affected. We also found similar relationships between the consequence variables as in Study 1, though the correlations were smaller and less significant.

Interestingly, the confidence manipulation was driven primarily by the effect of the manipulation designed to decrease confidence. Because the aim of our manipulation was to provide different levels of confidence in our participants—not to increase or decrease confidence *per se*—this does not impact the interpretation of our results. Nonetheless, the higher-confidence condition was closer to a control condition. In addition, although the vast majority of participants in the lower confidence condition were overconfident in their initial judgments and thus received feedback to reduce their confidence, not all of the participants did, because the advice provided to the participants depended on their initial confidence levels. Future work would thus benefit from having a stronger manipulation of confidence to provide a stronger differentiation between participants in the lower and higher confidence conditions.

## General discussion

Between the two studies, we showed that unjustified confidence influences psychological and behavioral variables as expected. Specifically, those with greater unjustified confidence displayed greater outcome expectations, lower anxiety, greater risk taking, and reduced information search and consideration. These relationships were demonstrated both correlationally (Study 1) and causally (Study 2), though the relationships were generally weaker and significant only by means of directional tests in Study 2.

**Figure 1:**
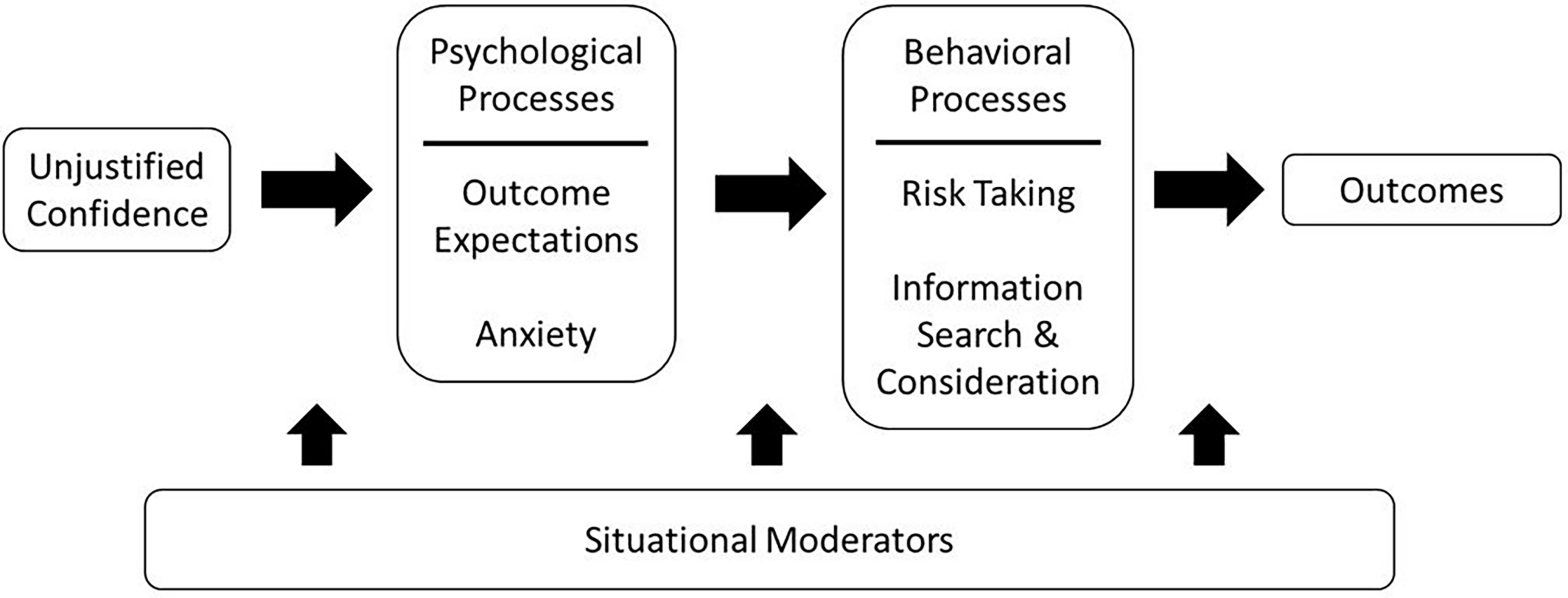
Proposed effects of unjustified confidence on psychological processes, behavioral processes, and subsequent decision outcomes.

Although only addressed here in exploratory analyses, we also found a number of relationships among the consequences themselves. It should be noted that we examined many different relationships, sometimes from an atheoretical perspective, and made no adjustments for capitalizing on chance. Thus, these findings should be considered tentative and future research would benefit from replicating these results. Additionally, this work would benefit from a causal examination of the consequences. Logically, the psychological consequences may occur first, selectively influencing the behavioral consequences, as depicted in Figure 1. In particular, we might expect that greater outcome expectations would promote greater risk taking. Heath and Tversky (1991), for example, argue that feelings of competence will lead to greater risk taking, in part due to outcome expectations. In contrast, higher state anxiety may inhibit risk taking, both for state anxiety (e.g., Raghunathan and Pham, 1999) and dispositional anxiety (e.g., Maner et al., 2007; Broman-Fulks et al., 2014). Anxiety has also been hypothesized to influence various types of information search and consideration (Raghunathan and Pham, 1999; Zhao and Cai, 2009; Lee and Hawkins, 2016). The correlations presented in Studies 1 and 2 provide further support for these three links, though our work does not provide evidence for the specific causal pathway.

### Limitations and boundary conditions

A strength of this research is that we examined a number of consequences within one particular domain (blackjack), thus providing an opportunity to examine a number of effects without their being confounded by domain. For example, we found that unjustified confidence reduced anxiety (presumably a good thing) but concurrently led to less willingness to use helpful outside information (presumably a bad thing) within the same context. Nonetheless, this approach leads to the primary limitation of this work—that any effects we found may be specific to the domain used, in this case, blackjack.

Indeed, blackjack, like any domain, has a number of unique characteristics. First, there are many ways in which cognitive biases impact blackjack gambling (e.g., Wagenaar, 1988). Although the aim of this research was not to investigate these biases, which are already well-studied, it is possible that they moderate the effect of confidence on our outcome measures. For example, our finding that confidence led to less use of the “hints for blackjack play” may have been strengthened by cognitive biases such as illusion of control (Langer, 1975; see also Wagenaar, 1988; Clark and Wohl, 2022). Second, the rules of blackjack and the implementation of the blackjack hints was straightforward. Thus one did not need to be confident in their ability to *use* the hints to improve their play; all one needed to do was follow the advice given to them. This situation is different from that studied in other work. For example, Utkarsh et al. (2019) found that participants with greater subjective knowledge were more apt to state they would obtain information about different models of smartphones, presumably because they believed they would be able to use the information effectively. Thus the link between confidence and information search and consideration may differ in situations where the available information is more challenging to use.

Further, blackjack is an example of a financial scenario. As discussed by Weber et al. (2002) and others, risk taking is relevant in a number of content domains outside the financial one, such as with health/safety risks and social risks, and it is plausible that some of the effects of confidence would vary by content domain. For example, in one recent study, Shou and Olney (2021) found that subjective knowledge had a stronger relationship with risk taking when risk information was implicit rather than explicit. Thus in situations where the risk is more ambiguous, it is possible that the effects would be stronger than the ones found in our study. Conversely, confidence appears to increase risk taking in situations where people believe they can use their knowledge to perform better, but if people do not believe that they can leverage their knowledge to produce better results, the confidence—risk taking link might disappear or even reverse (e.g., Lipkus et al., 2014).

One further limitation of this work is that we did not investigate the factors that produce unjustified confidence (i.e., the antecedents to unjustified confidence in Figure 1), instead taking confidence as a given (Study 1) or manipulating it in a manner distinct from what would happen in real-life (Study 2). Nonetheless, it is informative to compare our results to that of a paper by Phillips and Landon (2016). In their work, they manipulated whether participants had a winning streak or losing streak and found that participants who had a winning streak were more likely to bet more on subsequent trials and were less apt to take advice designed to improve play than were participants on a losing streak. Although Phillips and Landon did not directly measure confidence, combining the two studies suggests that past winning increases one’s confidence, which in turn leads one to bet more and be more resistant to taking helpful advice.

Finally, this research was about one type of confidence in particular, confidence in one’s knowledge. Confidence, however, can be about many different elements, such as one’s skilled performance (Harvey, 1994; Stone et al., 2011), one’s social competence (e.g., Li et al., 2020), or reflect a more general concept, such as self-efficacy (Bandura, 1977). These types of confidence are outside the scope of this investigation, and it is an open question as to whether these distinct types of confidence have similar or unique effects on the consequences studied in this work.

### Implications

A common lay theory states that a lack of confidence is a barrier to performance in many areas of life, such as athletic or scholastic performance. Indeed, a quick google search will bring up a very large number of sites extolling the virtues of confidence and providing advice on how to become more confident. Yet, within the judgment and decision making field, there is a great deal of evidence that people are overconfident in their knowledge, and this extends across levels of expertise and holds in most (though not all) domains (Yates, 1990). From this lens, increasing confidence without concurrently increasing knowledge will just increase overconfidence, essentially making a bad problem worse.

The aim of this research stream is to reconcile these views by understanding when confidence that is not justified by knowledge will produce positive vs. negative outcomes. Although other research has studied consequences of confidence, most of this work starts not with confidence, but rather with the outcome of interest (e.g., risk taking) and hence investigates confidence as one of many potential determinants of that outcome. Thus, that work is inherently limited to the consequence of interest in that work. Our work takes the opposite approach—starting with confidence and examining its effect on a number of different consequences. Our results suggest that there are in fact both positive and negative effects of increasing confidence. On the positive side, more confident people were less anxious. On the negative side, more confident people were less willing to consider additional information that would have helped their play. Other effects are more value-neutral, such as with the link between confidence and risk taking. Within blackjack specifically, risk taking would be negative (since most people lose money), but in other situations an increased willingness to take risks would be beneficial.

More generally, this research stream points to the need to consider the ramifications of situations when confidence increases independent of knowledge. Phillips and Landon’s (2016) work showing that winning in blackjack can cause an increase in betting and less willingness to take blackjack advice provides a striking demonstration of this concern; even a positive outcome can have a long-term negative impact under certain circumstances. Note this concern holds even for situations where knowledge increases, but at a rate less than the increase in confidence. One potential example is the use of educational interventions, such as teaching the public about the home risks of radon or educational efforts to increase financial capability. On the surface, the logic is compelling—if we teach people the facts, they will make more informed (and hopefully better) decisions. However, we know that informational interventions tend to increase confidence, regardless of whether they increase knowledge (see Oskamp, 1965; Stone and Opel, 2000). Given the potential negative consequences of unjustified confidence, this is concerning. For example, Jaccard et al. (2005) suggest that informing adolescents about risk behavior and potential ways of avoiding negative outcomes has the potential to produce adverse effects if the message increases the adolescents’ perceived ability without increasing actual ability sufficiently.

To address these concerns, education targeting participants’ confidence levels as well as their knowledge could be provided. Smith and Dumont (1997), for example, recommend including methods to reduce student overconfidence levels within clinical psychology training programs. To the extent that such domains experience negative outcomes of unjustified confidence, this type of advice seems warranted. Nonetheless, this advice is predicated on the assumption that unjustified confidence has negative effects; if the main impact of correcting students’ confidence was to increase their anxiety levels, for example, even this advice could backfire.

The overall goal of this work was to provide empirical evidence of the effects of confidence on a range of different psychological and behavioral variables, but the consequences studied here are far from comprehensive. Further research should extend this work to other consequences, including to additional domains where different consequences would be relevant. Ultimately, studies should also seek to establish how these psychological and behavioral consequences relate to real-world outcomes. The current studies were not powered to detect effects on amount won or lost (in blackjack it would have required a much longer period of play to establish stable estimates of outcome success); however, we did examine the relationship between viewing of the hints for blackjack play and quality of play as operationalized as decisions in keeping with basic strategy (Thorp, 1966), as it is well-established that making decisions in keeping with basic strategy produces better outcomes. In particular, in Study 2 where we had behavioral data, we found that play quality was positively correlated with number of times the hints were viewed (*r* = 0.43) and the amount of time the hints were viewed for (*r* = 0.45). This finding is in keeping with past studies suggesting that there is an impact of unjustified confidence on real-world outcomes (e.g., Price and Stone, 2004; Parker and Fischhoff, 2005; Parker et al., 2012; Hadar et al., 2013; Parker and Stone, 2014).

## Data availability statement

The raw data supporting the conclusions of this article will be made available by the authors, without undue reservation.

## Ethics statement

The studies involving human participants were reviewed and approved by Wake Forest Institutional Review Board. The patients/participants provided their written informed consent to participate in this study.

## Author contributions

ES and AP were involved in the study design, data analyses, and writing. AH conducted the studies and assisted in writing the manuscript. RS wrote the blackjack program and assisted in other ways as required. All authors contributed to the article and approved the submitted version.

## Funding

At Wake Forest, open access publication is split 1/3 (library), 1/3 (office of sponsored research), and 1/3 (primary investigator or their department).

## Conflict of interest

AP was employed by RAND Corporation. AH was employed by Talogy. RS was employed by Allego, Inc.

The remaining authors declare that the research was conducted in the absence of any commercial or financial relationships that could be construed as a potential conflict of interest.

## Publisher’s note

All claims expressed in this article are solely those of the authors and do not necessarily represent those of their affiliated organizations, or those of the publisher, the editors and the reviewers. Any product that may be evaluated in this article, or claim that may be made by its manufacturer, is not guaranteed or endorsed by the publisher.

## Figures and Tables

**Table:** 

	Anx.	Avg. Bet	ISC
Outcome expectations	−0.13	0.28	−0.11
Anxiety	−	−0.19	0.23
Average bet		−	−0.04
Information Search and Consideration			−

**Table:** 

	Anx.	Avg. Bet	ISC
Outcome expectations	−1.89	4.10	−1.58
Anxiety	−	−2.69	3.21
Average bet		−	−0.50
Information Search and Consideration			−

## Footnotes

^1^Our description of overconfidence here most closely reflects a combination of what Moore and Healy (2008) call “overestimation” and “overprecision.”

^2^Due to the inherent noisiness in blackjack outcomes, it would take a much longer series of gambles to observe reliable effects on decision outcomes (e.g., winnings). That said, in both Studies 1 and 2 we conducted exploratory analyses, the results of which (not reported here) were largely non-significant but consistent with the idea that the behavioral changes produced downstream effects on decision outcomes.

^3^In retrospect, the wording of this question (“Did you ever disregard the blackjack suggestions?”) was awkward and double-barreled (no could mean not looking at the suggestions or not rejecting once looking), making the lack of a strong positive correlation unsurprising.

^4^The second version was constructed in the same manner as the original version and was constrained to match the original version as closely as possible. For example, in the original version participants were asked three questions about whether they would hit or stand when they had a point total of 13, assuming a specific up-card on the part of the dealer. Thus, in this second version of the KCA, participants were asked three (different) questions regarding whether they would hit or stand when they had a point total of 13.

^5^The number of items answered correctly and incorrectly per each confidence judgment category (e.g., 80%) was entered into an Excel spreadsheet, which created the participant’s calibration graph based on these data. A problem with this program was discovered approximately one-third through the study; if a confidence judgment was not given, the program reported that 0% of the judgments at that interval were correct, rather than omitting that rating interval from the calibration graph. Thus, in these cases, the graph showed that participants answered 0% of the items correctly when in fact they had not made any confidence judgments for that rating interval. In the remaining sessions, the experimenter corrected the graphs when necessary to reflect that the confidence interval had not been used. That this problem had minimal if any effect on the manipulation is shown by the successful manipulation check, as documented subsequently.

## Appendix

To combine the correlations among the consequences in Studies 1 and 2, we used the procedure recommended by Goh et al. (2016) for combining results of studies within a manuscript. The procedure works as follows:

**Step 1: Compute Average Correlation Across the Studies.**

Begin by converting each of the correlations (shown in Tables 1, 2) through Fisher’s transformation. Then, average these transformed correlations (r_z_) *via* the following formula:

$$\text{Weighted}{\bar{r}}_{z}=\frac{\sum \left(\left[N-3\right]r_{z}\right)}{\sum \left(N-3\right)}$$

Then, transform back to an *r* to have the results in the more easily recognizable *r* metric. These combined *r*’s are shown in the table below. Note that these *r*’s are very similar to what would have been produced by doing a straight average of the correlation coefficients, but are slightly different due to having used Fisher’s transformation.

Correlations Among the Consequences Combined Across Studies 1 and 2.

**Step 2: Perform an Overall Test of Significance Across the Studies.**

Begin by calculating z’s for each of the relationships in each of the studies. Goh et al. provide the following formula when one has correlations:

$$Z=r\sqrt{N}$$

Then, use the following formula to produce a combined z, where k is the number of studies:

$$Z_{\text{combined}}=\frac{\sum N}{\sqrt{k}}$$

Using this procedure produces the following table of *z* values:

Combined Values of *z* for Testing Significance Combined Across Studies 1 and 2

From there, it’s straightforward to convert the *z* to its value of *p* using the normal table or a computer program.
