# Supplementary material for: Thinking without knowing: Psychological and behavioral consequences of unjustified confidence regarding blackjack strategy
Source: Front Psychol. 2023 Jan 26;14:1015676. doi: 10.3389/fpsyg.2023.1015676 (PMC9909440; doi:10.3389/fpsyg.2023.1015676)
Supplement: Supplementary file 1 [file Data_Sheet_1.PDF]

Supplementary Materials for "Thinking without knowing: Psychological and behavioral consequences of unjustified confidence regarding blackjack strategy"

Eric R. Stone, Andrew M. Parker, Ashley Rittmayer Hanks, & Robert C. Swiston

Goals of the Supplementary Material

The goal of these supplementary materials is to provide additional information about our methods. Specifically, we (1) describe the confidence manipulation in more detail than described in the text, and (2) provide the full versions of each of our questionnaire measures.

Study 2 Confidence Manipulation

Lower Confidence

As discussed in the main text, this condition involved providing participants with their personal calibration diagram and a brief one-on-one feedback session. (Recall that all participants had already received a lecture on the concept of calibration prior to filling out the first Knowledge-Confidence Assessment.) This session consisted of an explanation of the calibration graph, as well as suggestions for improvement. Particular emphasis was placed on avoiding the use of extreme categories, when appropriate. For example, the experimenter might explain that the calibration diagram indicates overconfidence due to the points being below the line of perfect calibration, adding that out of all the cases the participant said they were 100% sure, they were correct only x% of the time. The experimenter would then offer advice for improvement, in this case, that to become better calibrated the participant should be less confident and especially to express very high confidence less frequently. The information conveyed always was truthful. As mentioned in the main text, this individualized feedback was designed for producing better calibration, but since the vast majority of participants were overconfident, it serves as a measure of decreasing confidence as well.

At the conclusion of the one-on-one feedback session, the experimenter asked each participant to restate the calibration advice. In most cases, this entailed an acknowledgement of the need to be less confident. After all participants in the session received the manipulation, the experimenter gave each participant their calibration graph to review for approximately two minutes before completing the second knowledge-confidence assessment.

Higher Confidence

As discussed in the main text, this condition involved providing participants with information that appeared to be helpful, but was not actually useful for improving blackjack play. The approach was modeled after Oskamp's (1965) research showing that as psychologists accumulated additional information, their confidence in their ability to make accurate diagnostic conclusions increased at a rate not justified by an actual increase in diagnostic ability. For this study, we provided participants a graph of "The odds of possible outcomes, depending on the player's hand." On the x-axis, this graph depicted the different player hands (e.g., 10, 11, 12). The y-axis then provided the odds of different outcomes after

drawing one more card, with green points indicating the probability of busting, yellow points the probability of having a hand between 17-21, and pink points the odds of having a hand under 17. For example, if the player had a hand of "11" (say due to a 7 and a 4), then the chart would say there was a 0% chance of busting, a 5/13 chance of ending with a hand under 17, and an 8/13 chance of ending with a hand between 17 and 21 after drawing an additional card. Knowing these odds should make participants feel more knowledgeable about the task, but should not actually improve their play since it is not clear how to use this knowledge.

As in the lower confidence condition, the experimenter conducted a brief one-on-one session with each participant, which included an explanation of the blackjack odds graph and encouragement that this information should be helpful when making judgments regarding blackjack play. After participants received this information, they were asked if they had any questions regarding the information. As in the lower confidence condition, once all participants in the session received the manipulation, the experimenter gave each participant a copy of the graph to review for approximately two minutes before completing the second knowledge-confidence assessment.

Measures

Study 1

Knowledge-Confidence Assessment.

Participants were provided with 40 items asking about their knowledge of the proper play covering four areas of blackjack play: when to hit versus stand, split, double down, and buy insurance. For each item, participants provided both an estimate of which answer was correct as well as a confidence judgment. The precise instructions and items were as follows:

INSTRUCTIONS

This survey presents casino blackjack hands for which you must decide which of two options you should choose, such as "Hit or Stand" or "Split or Not," to maximize your earnings. "You" represents the cards in your (the player's) hand. For the "Dealer" you are shown only the dealer's upcard, though the dealer's hand includes another card that is face down. For example,

You: 3, 4 **Dealer: Q**

To maximize your earnings, should you?: Hit or Stand

We want you to do two things for each item.

First, answer the question. In this example, you might think "*Since I can't go over 21, I should hit."* Then you would circle 'Hit.'

You: 3, 4 **Dealer: Q**

To maximize your earnings, should you?: Hit or Stand

Second, think about how sure you are of your answer. Give a number from 50% to 100%. In other words, what is the percent chance that you are right? Circle one of the numbers on the scale.

50% 60% 70% 80% 90% 100%
just guessing absolutely sure

If your answer is a total guess, circle 50%. This means that there is a 50% chance that you are right, and a 50% chance that you are wrong. If you are absolutely sure, circle 100%. If you aren't sure, then circle a number in between, to show how sure you are.

In the above example, you might think "*I am absolutely sure I should hit, so 100%*." So, you would circle 100%.

You: 3, 4 **Dealer: Q**

To maximize your earnings, should you? Hit or Stand

50% 60% 70% 80% 90% 100%
just guessing absolutely sure

Now let's say that you had no idea what to do in the situation. You would then make a random choice between the options.

Your answer would look like this:

<u>You</u>: 3, 4 **<u>Dealer</u>: Q**

To maximize your earnings, should you? Hit or Stand

50% 60% 70% 80% 90% 100%
just guessing absolutely sure

In many cases you will have some idea what you should do, but you won't be certain. In these situations, give a number somewhere between 50% and 100%, depending on how sure you are. For example,

<u>You</u>: 3, 4 **<u>Dealer</u>: Q**

To maximize your earnings, should you? Hit or Stand

50% 60% 70% 80% 90% 100%
just guessing absolutely sure

Or, if you were 60% sure, you would do the following:

<u>You</u>: 3, 4 **<u>Dealer</u>: Q**

To maximize your earnings, should you? Hit or Stand

50% 60% 70% 80% 90% 100%
just guessing absolutely sure

Next, you will be presented with 40 questions that ask which of two decisions should be made regarding different aspects of blackjack play. Please answer these questions the same way as shown in these examples. If you have any questions, please ask the experimenter now.

<u>Part I: Hit or Stand</u>

For each of these situations you will be provided with your hand and the dealer's upcard and asked whether you should hit or stand to maximize your earnings. For each item circle **Hit** or **Stand** and the probability that you are correct.

1. **<u>You</u>: 10, 7** **<u>Dealer</u>: 10**

To maximize your earnings, should you?: Hit or Stand

50%	60%	70%	80%	90%	100%
just guessing					absolutely sure

2. **<u>You</u>: 10, 2** **<u>Dealer</u>: 8**

To maximize your earnings, should you?: Hit or Stand

50%	60%	70%	80%	90%	100%
just guessing					absolutely sure

3. **<u>You</u>: 9, 5** **<u>Dealer</u>: 7**

To maximize your earnings, should you?: Hit or Stand

50%	60%	70%	80%	90%	100%
just guessing					absolutely sure

4. **<u>You</u>: 10, 2** **<u>Dealer</u>: 7**

To maximize your earnings, should you?: Hit or Stand

50%	60%	70%	80%	90%	100%
just guessing					absolutely sure

5. **<u>You</u>: 9, 7** **<u>Dealer</u>: 6**

To maximize your earnings, should you?: Hit or Stand

50%	60%	70%	80%	90%	100%
just guessing					absolutely sure

6. **<u>You</u>: 3, K** **<u>Dealer</u>: 2**

To maximize your earnings, should you?: Hit or Stand

50%	60%	70%	80%	90%	100%
just guessing					absolutely sure

7. **<u>You</u>: K, 6** **<u>Dealer</u>: 5**

To maximize your earnings, should you?: Hit or Stand

50%	60%	70%	80%	90%	100%
just guessing					absolutely sure

8. **<u>You</u>: 4, K** **<u>Dealer</u>: 2**

To maximize your earnings, should you?: Hit or Stand

50%	60%	70%	80%	90%	100%
just guessing					absolutely sure

9. **<u>You</u>: A, 7** **<u>Dealer</u>: 7**

To maximize your earnings, should you?: Hit or Stand

50%	60%	70%	80%	90%	100%
just guessing					absolutely sure

10. **<u>You</u>: J, 6** **<u>Dealer</u>: 7**

To maximize your earnings, should you?: Hit or Stand

50%	60%	70%	80%	90%	100%
just guessing					absolutely sure

11. <u>You</u>: 7, 5 **<u>Dealer</u>: 6**

To maximize your earnings, should you?: Hit or Stand

50%	60%	70%	80%	90%	100%
just guessing					absolutely sure

12. <u>You</u>: 10, 5 **<u>Dealer</u>: 4**

To maximize your earnings, should you?: Hit or Stand

50%	60%	70%	80%	90%	100%
just guessing					absolutely sure

13. <u>You</u>: 10, 2 **<u>Dealer</u>: 3**

To maximize your earnings, should you?: Hit or Stand

50%	60%	70%	80%	90%	100%
just guessing					absolutely sure

14. <u>You</u>: 10, 5 **<u>Dealer</u>: 3**

To maximize your earnings, should you?: Hit or Stand

50%	60%	70%	80%	90%	100%
just guessing					absolutely sure

15. <u>You</u>: 8, 7 **<u>Dealer</u>: 8**

To maximize your earnings, should you?: Hit or Stand

50%	60%	70%	80%	90%	100%
just guessing					absolutely sure

16. <u>You</u>: 5, 10 **<u>Dealer</u>: 5**

To maximize your earnings, should you?: Hit or Stand

50%	60%	70%	80%	90%	100%
just guessing					absolutely sure

17. <u>You</u>: A, 7 **<u>Dealer</u>: K**

To maximize your earnings, should you?: Hit or Stand

50%	60%	70%	80%	90%	100%
just guessing					absolutely sure

18. <u>You</u>: 4, 10 **<u>Dealer</u>: 8**

To maximize your earnings, should you?: Hit or Stand

50%	60%	70%	80%	90%	100%
just guessing					absolutely sure

19. <u>You</u>: 3, K **<u>Dealer</u>: 7**

To maximize your earnings, should you?: Hit or Stand

50%	60%	70%	80%	90%	100%
just guessing					absolutely sure

20. <u>You</u>: J, 3 **<u>Dealer</u>: 4**

To maximize your earnings, should you?: Hit or Stand

50%	60%	70%	80%	90%	100%
just guessing					absolutely sure

Part II: Double down or Not

For each of these situations you will be provided with your hand and the dealer's upcard and asked whether you should double down or not to maximize your earnings. For each item circle **Yes** or **No** and the probability that you are correct.

21. **You**: A, 5 **Dealer**: 6

To maximize your earnings, should you double down?: Yes or No

50%	60%	70%	80%	90%	100%
just guessing					absolutely sure

22. **You**: 5, 4 **Dealer**: 9

To maximize your earnings, should you double down?: Yes or No

50%	60%	70%	80%	90%	100%
just guessing					absolutely sure

23. **You**: 9, 2 **Dealer**: 6

To maximize your earnings, should you double down?: Yes or No

50%	60%	70%	80%	90%	100%
just guessing					absolutely sure

24. **You**: 9, 2 **Dealer**: J

To maximize your earnings, should you double down?: Yes or No

50%	60%	70%	80%	90%	100%
just guessing					absolutely sure

25. **You**: 7, 3 **Dealer**: 2

To maximize your earnings, should you double down?: Yes or No

50%	60%	70%	80%	90%	100%
just guessing					absolutely sure

26. **You**: A, 5 **Dealer**: 9

To maximize your earnings, should you double down?: Yes or No

50%	60%	70%	80%	90%	100%
just guessing					absolutely sure

27. <u>You</u>: 5, 4 **<u>Dealer</u>: 2**

To maximize your earnings, should you double down?: Yes or No

50%	60%	70%	80%	90%	100%
just guessing					absolutely sure

28. <u>You</u>: A, 2 **<u>Dealer</u>: J**

To maximize your earnings, should you double down?: Yes or No

50%	60%	70%	80%	90%	100%
just guessing					absolutely sure

29. <u>You</u>: 7, 3 **<u>Dealer</u>: K**

To maximize your earnings, should you double down?: Yes or No

50%	60%	70%	80%	90%	100%
just guessing					absolutely sure

30. <u>You</u>: A, 2 **<u>Dealer</u>: 6**

To maximize your earnings, should you double down?: Yes or No

50%	60%	70%	80%	90%	100%
just guessing					absolutely sure

Part III: Split or Not

For each of these situations you will be provided with your hand and the dealer's upcard and asked whether you should split your cards or not to maximize your earnings. For each item circle **Yes** or **No** and the probability that you are correct.

31. You: A, A **Dealer: 3**

To maximize your earnings, should you split your cards or not?: Yes or No

50% just guessing	60%	70%	80%	90%	100% absolutely sure

32. You: 9, 9 **Dealer: 8**

To maximize your earnings, should you split your cards or not?: Yes or No

50% just guessing	60%	70%	80%	90%	100% absolutely sure

33. You: K, K **Dealer: 6**

To maximize your earnings, should you split your cards or not?: Yes or No

50% just guessing	60%	70%	80%	90%	100% absolutely sure

34. You: 2, 2 **Dealer: Q**

To maximize your earnings, should you split your cards or not?: Yes or No

50% just guessing	60%	70%	80%	90%	100% absolutely sure

35. You: 7, 7 **Dealer: 4**

To maximize your earnings, should you split your cards or not?: Yes or No

50% just guessing	60%	70%	80%	90%	100% absolutely sure

36. You: 4, 4 **Dealer: 9**

To maximize your earnings, should you split your cards or not?: Yes or No

50% 60% 70% 80% 90% 100%
just guessing absolutely sure

Part IV: Insurance or Not

For each of these situations you will be provided with your hand and the dealer's upcard and asked whether you should buy insurance or not to maximize your earnings. For each item circle **Yes** or **No** and the probability that you are correct.

37. You: 5, Q **Dealer: A**

To maximize your earnings, should you buy insurance or not?: Yes or No

50% 60% 70% 80% 90% 100%
just guessing absolutely sure

38. You: 4, 5 **Dealer: A**

To maximize your earnings, should you buy insurance or not?: Yes or No

50% 60% 70% 80% 90% 100%
just guessing absolutely sure

39. You: A, J **Dealer: A**

To maximize your earnings, should you buy insurance or not?: Yes or No

50% 60% 70% 80% 90% 100%
just guessing absolutely sure

40. You: 9, J **Dealer: A**

To maximize your earnings, should you buy insurance or not?: Yes or No

50% 60% 70% 80% 90% 100%
just guessing absolutely sure

Outcome expectations.

Outcome expectations was measured by two questions, both given prior to performing the main task:

1. What is the likelihood that you will win more than the average person during today's blackjack game?

0% 10% 20% 30% 40% 50% 60% 70% 80% 90% 100%

2. What is the likelihood that you will win more money than you lose during today's blackjack game?

0% 10% 20% 30% 40% 50% 60% 70% 80% 90% 100%

These items were averaged to form our measure of outcome expectations.

Anxiety.

Anxiety was measured by the State-Trait Anxiety Inventory (STAI; Spielberger, Gorsuch & Luschene, 1970). The items and instructions are as follows:

A number of statements which people have used to describe themselves are given below. Read each statement and then circle the appropriate number to the right of the statement to indicate how you felt *while playing blackjack*, that is, *during today's experiment*. There are no right or wrong answers. Do not spend too much time on any one statement but give the answer that seems to describe the way you felt best.

Answer choices are (**1**) **almost never**, (**2**) **sometimes**, (**3**) **often**, or (**4**) **almost always**.

1. I felt calm.
1 2 3 4

2. I felt secure.
1 2 3 4

3. I was tense.
1 2 3 4

4. I felt strained.
1 2 3 4

5. I felt at ease.
1 2 3 4

6. I felt upset.
1 2 3 4

7. I was worried over possible misfortunes.
1 2 3 4

8. I felt satisfied.
1 2 3 4

9. I felt frightened.
1 2 3 4

10. I felt comfortable.
1 2 3 4

11. I felt self-confident.
1 2 3 4

12. I felt nervous.
1 2 3 4

13. I was jittery.
1 2 3 4

14. I felt indecisive.
1 2 3 4

15. I was relaxed.
1 2 3 4

16. I felt content.
1 2 3 4

17. I was worried.
1 2 3 4

18. I felt confused.
1 2 3 4

19. I felt steady.
1 2 3 4

20. I felt pleasant.
1 2 3 4

We averaged responses to the above 20 items, reverse scoring when appropriate.

Information search and consideration.

At the conclusion of the study, participants were provided 9 items asking about the extent to which they used hints about good blackjack play provided to them and would want additional information if playing casino blackjack in the future. These items were:

1. How often did you look at the blackjack suggestions?

1	2	3	4	5
not at all	occasionally	sometimes	quite often	very frequently

2. How often did you follow the blackjack suggestions?

1	2	3	4	5
not at all	occasionally	sometimes	quite often	very frequently

3. How many times did you review the blackjack suggestions *before your hand was dealt*?

0 1-2 3-5 6-10 more than 10

4. How many times did you consult the blackjack suggestions *after getting your cards* in order to decide what to do?

0 1-2 3-5 6-10 more than 10

[1]5. Did you ever change your blackjack play based on the suggestions? Yes or No

If yes, how many times? _______

[2]6. Did you ever disregard the blackjack suggestions? Yes or No

If yes, how many times? _______

7. All told, how useful did you find the blackjack suggestions to be?

1	2	3	4	5	6	7
not at all useful			moderately useful			very useful

If you were to play blackjack in an actual casino….

1. Would you want to consult a book or person for additional suggestions on strategy?

1	2	3	4	5	6	7
definitely no			maybe			definitely yes

[3]2. Would you tend to follow expert advice or rely on your intuition?

1	2	3	4	5	6	7
solely follow expert advice			equally follow advice and intuition			solely follow intuition

Notes:

[1] We only scored whether they ever changed their blackjack play or not (not how many times). This was coded such that yes was a higher number.

[2] As discussed in the main text, after recoding, this item still did not correlate with the other items, so we eliminated it from our scale.

[3] This item was reverse scored.

Study 2

Knowledge-Confidence Assessment.

As discussed in the main text, participants took two versions of this questionnaire in Study 2. One version was identical to that used in Study 1. The other version is as follows:

<u>Part I: Hit or Stand</u>

For each of these situations you will be provided with your hand and the dealer's upcard and asked whether you should hit or stand to maximize your earnings. For each item circle **Hit** or **Stand** and the probability that you are correct.

1. **<u>You</u>: Q, 5** **<u>Dealer</u>: Q**

To maximize your earnings, should you?: Hit or Stand

50%	60%	70%	80%	90%	100%
just guessing					absolutely sure

2. **<u>You</u>: A, 7** **<u>Dealer</u>: 2**

To maximize your earnings, should you?: Hit or Stand

50%	60%	70%	80%	90%	100%

just guessing absolutely sure

3. **You**: **9, 6** **Dealer**: **6**

To maximize your earnings, should you?: Hit or Stand

50% 60% 70% 80% 90% 100%
just guessing absolutely sure

4. **You**: **Q, 3** **Dealer**: **8**

To maximize your earnings, should you?: Hit or Stand

50% 60% 70% 80% 90% 100%
just guessing absolutely sure

5. **You**: **4, J** **Dealer**: **K**

To maximize your earnings, should you?: Hit or Stand

50% 60% 70% 80% 90% 100%
just guessing absolutely sure

6. **You**: **J, 4** **Dealer**: **4**

To maximize your earnings, should you?: Hit or Stand

50% 60% 70% 80% 90% 100%
just guessing absolutely sure

7. **You**: **A, 7** **Dealer**: **8**

To maximize your earnings, should you?: Hit or Stand

50% 60% 70% 80% 90% 100%
just guessing absolutely sure

8. **You**: **4, 9** **Dealer**: **3**

To maximize your earnings, should you?: Hit or Stand

50% 60% 70% 80% 90% 100%
just guessing absolutely sure

9. <u>You</u>: K, 6 **<u>Dealer</u>: 8**

To maximize your earnings, should you?: Hit or Stand

50%	60%	70%	80%	90%	100%
just guessing					absolutely sure

10. <u>You</u>: 9, 4 **<u>Dealer</u>: 9**

To maximize your earnings, should you?: Hit or Stand

50%	60%	70%	80%	90%	100%
just guessing					absolutely sure

11. <u>You</u>: Q, 6 **<u>Dealer</u>: 2**

To maximize your earnings, should you?: Hit or Stand

50%	60%	70%	80%	90%	100%
just guessing					absolutely sure

12. <u>You</u>: 4, 8 **<u>Dealer</u>: 5**

To maximize your earnings, should you?: Hit or Stand

50%	60%	70%	80%	90%	100%
just guessing					absolutely sure

13. <u>You</u>: 9, 3 **<u>Dealer</u>: K**

To maximize your earnings, should you?: Hit or Stand

50%	60%	70%	80%	90%	100%
just guessing					absolutely sure

14. <u>You</u>: J, 5 **<u>Dealer</u>: 2**

To maximize your earnings, should you?: Hit or Stand

50%	60%	70%	80%	90%	100%
just guessing					absolutely sure

15. <u>You</u>: 9, 5 **<u>Dealer</u>: 2**

To maximize your earnings, should you?: Hit or Stand

50% 60% 70% 80% 90% 100%
just guessing absolutely sure

16. <u>You</u>: 2, Q **<u>Dealer</u>: 9**

To maximize your earnings, should you?: Hit or Stand

50% 60% 70% 80% 90% 100%
just guessing absolutely sure

17. <u>You</u>: 7, 9 **<u>Dealer</u>: 4**

To maximize your earnings, should you?: Hit or Stand

50% 60% 70% 80% 90% 100%
just guessing absolutely sure

18. <u>You</u>: 7, 10 **<u>Dealer</u>: 8**

To maximize your earnings, should you?: Hit or Stand

50% 60% 70% 80% 90% 100%
just guessing absolutely sure

19. <u>You</u>: J, 2 **<u>Dealer</u>: 2**

To maximize your earnings, should you?: Hit or Stand

50% 60% 70% 80% 90% 100%
just guessing absolutely sure

20. <u>You</u>: 7, 8 **<u>Dealer</u>: 6**

To maximize your earnings, should you?: Hit or Stand

50% 60% 70% 80% 90% 100%
just guessing absolutely sure

<u>Part II: Double down or Not</u>

For each of these situations you will be provided with your hand and the dealer's upcard and asked whether you should double down or not to maximize your earnings. For each item circle **Yes** or **No** and the probability that you are correct.

21. <u>You</u>: A, 2 **<u>Dealer</u>: 3**

To maximize your earnings, should you double down?: Yes or No

50% 60% 70% 80% 90% 100%
just guessing absolutely sure

22. <u>You</u>: 9, 2 **<u>Dealer</u>: 4**

To maximize your earnings, should you double down?: Yes or No

50% 60% 70% 80% 90% 100%
just guessing absolutely sure

23. <u>You</u>: 5, 4 **<u>Dealer</u>: Q**

To maximize your earnings, should you double down?: Yes or No

50% 60% 70% 80% 90% 100%
just guessing absolutely sure

24. <u>You</u>: 7, 3 **<u>Dealer</u>: A**

To maximize your earnings, should you double down?: Yes or No

50% 60% 70% 80% 90% 100%
just guessing absolutely sure

25. <u>You</u>: A, 5 **<u>Dealer</u>: 3**

To maximize your earnings, should you double down?: Yes or No

50% 60% 70% 80% 90% 100%
just guessing absolutely sure

26. <u>You</u>: 7, 3 **<u>Dealer</u>: 4**

To maximize your earnings, should you double down?: Yes or No

50% 60% 70% 80% 90% 100%
just guessing absolutely sure

27. <u>You</u>: 5, 4 **<u>Dealer</u>: 3**

To maximize your earnings, should you double down?: Yes or No

50%	60%	70%	80%	90%	100%
just guessing					absolutely sure

28. <u>You</u>: A, 2 **<u>Dealer</u>: 8**

To maximize your earnings, should you double down?: Yes or No

50%	60%	70%	80%	90%	100%
just guessing					absolutely sure

29. <u>You</u>: 9, 2 **<u>Dealer</u>: 9**

To maximize your earnings, should you double down?: Yes or No

50%	60%	70%	80%	90%	100%
just guessing					absolutely sure

30. <u>You</u>: A, 5 **<u>Dealer</u>: 8**

To maximize your earnings, should you double down?: Yes or No

50%	60%	70%	80%	90%	100%
just guessing					absolutely sure

<u>Part III: Split or Not</u>

For each of these situations you will be provided with your hand and the dealer's upcard and asked whether you should split your cards or not to maximize your earnings. For each item circle **Yes** or **No** and the probability that you are correct.

31. <u>You</u>: 9, 9 **<u>Dealer</u>: A**

To maximize your earnings, should you split your cards or not?: Yes or No

50%	60%	70%	80%	90%	100%
just guessing					absolutely sure

32. <u>You</u>: 7, 7 **<u>Dealer</u>: 7**

To maximize your earnings, should you split your cards or not?: Yes or No

50%	60%	70%	80%	90%	100%
just guessing					absolutely sure

33. <u>You</u>: 2, 2 **<u>Dealer</u>: 3**

To maximize your earnings, should you split your cards or not?: Yes or No

50%	60%	70%	80%	90%	100%
just guessing					absolutely sure

34. <u>You</u>: A, A **<u>Dealer</u>: 9**

To maximize your earnings, should you split your cards or not?: Yes or No

50%	60%	70%	80%	90%	100%
just guessing					absolutely sure

35. <u>You</u>: 4, 4 **<u>Dealer</u>: 6**

To maximize your earnings, should you split your cards or not?: Yes or No

50%	60%	70%	80%	90%	100%
just guessing					absolutely sure

36. <u>You</u>: K, K **<u>Dealer</u>: 5**

To maximize your earnings, should you split your cards or not?: Yes or No

50%	60%	70%	80%	90%	100%
just guessing					absolutely sure

<u>Part IV: Insurance or Not</u>

For each of these situations you will be provided with your hand and the dealer's upcard and asked whether you should buy insurance or not to maximize your earnings. For each item circle **Yes** or **No** and the probability that you are correct.

37. <u>You</u>: Q, 6 **<u>Dealer</u>: A**

To maximize your earnings, should you buy insurance or not?: Yes or No

50%	60%	70%	80%	90%	100%
just guessing					absolutely sure

38. <u>You</u>: 8, 9 **<u>Dealer</u>: A**

To maximize your earnings, should you buy insurance or not?: Yes or No

50%	60%	70%	80%	90%	100%
just guessing					absolutely sure

39. <u>You</u>: 3, 4 **<u>Dealer</u>: A**

To maximize your earnings, should you buy insurance or not?: Yes or No

50%	60%	70%	80%	90%	100%
just guessing					absolutely sure

40. <u>You</u>: J, 10 **<u>Dealer</u>: A**

To maximize your earnings, should you buy insurance or not?: Yes or No

50%	60%	70%	80%	90%	100%
just guessing					absolutely sure

Outcome expectations.

Outcome expectations was measured by four questions, all given prior to performing the main task:

The following two questions ask about your perceptions of <u>winning</u>…

1. What is the likelihood that you will ***win*** *more than you lose* during today's blackjack game?

   0% 10% 20% 30% 40% 50% 60% 70% 80% 90% 100%

2. How pleased would you be if you ***win*** *more than you lose* during today's blackjack game?

1	2	3	4	5	6	7
not at all (indifferent)			moderately			extremely

The following two questions ask about your perceptions of <u>losing</u>…

3. What is the likelihood that you will ***lose*** *more than you win* during today's blackjack game?

   0% 10% 20% 30% 40% 50% 60% 70% 80% 90% 100%

4. How disappointed would you be if you ***lose*** *more money than you win* during today's blackjack game?

1	2	3	4	5	6	7
not at all (indifferent)			moderately			extremely

Anxiety.

Anxiety was measured via the same items used in Study 1. However, as discussed in the main text, we changed the response scale. The instructions to this scale now read:

A number of statements which people have used to describe themselves are given below. Read each statement and then circle the appropriate number to the right of the statement to indicate how you felt *while playing blackjack*, that is, *during today's experiment*. There are no right or wrong answers. Do not spend too much time on any one statement but give the answer that seems to describe the way you felt best.

Answer choices are (**1**) **not at all**, (**2**) **somewhat**, (**3**) **moderately so**, or (**4**) **very much so**.

Information search and consideration (self-report measure).

As discussed in the main text, we modified the self-report measure to be more in keeping with the way the information was displayed in this study. The items were:

1. How often did you look at the blackjack suggestions?

1	2	3	4	5
not at all	occasionally	sometimes	quite often	very frequently

2. How often did you follow the blackjack suggestions?

1	2	3	4	5
not at all	occasionally	sometimes	quite often	very frequently

3. Did you ever change your blackjack play based on the suggestions? Yes or No

   If yes, how many times? _______

4. Did you ever disregard the blackjack suggestions? Yes or No

   If yes, how many times? _______

5. All told, how useful did you find the blackjack suggestions to be?

1	2	3	4	5	6	7
not at all useful			moderately useful			very useful

If you were to play blackjack in an actual casino….

1. Would you want to consult a book or person for additional suggestions on strategy?

1	2	3	4	5	6	7
definitely no			maybe			definitely yes

2. Would you tend to follow expert advice or rely on your intuition?

1	2	3	4	5	6	7
solely follow expert advice			equally follow advice and intuition			solely follow intuition

The items were scored as in Study 1 (i.e., the same items were reverse scored, and we did not consider the number of times the player changed their play based on the suggestions, just whether or not they always did.) Also as in Study 1, we eliminated the item about disregarding the blackjack suggestions, as it again had a negative item-total correlation.
